# Author Correction: Adherence to public institutions that foster cooperation

**DOI:** 10.1038/s41467-021-24338-8

**Published:** 2021-07-21

**Authors:** Arunas L. Radzvilavicius, Taylor A. Kessinger, Joshua B. Plotkin

**Affiliations:** Department of Biology, University of Pennsylvania, Philadelphia, PA USA

**Keywords:** Social evolution, Society

Correction to: *Nature Communications* 10.1038/s41467-021-23783-9

The original version of this Article contained an error in Fig 2., in which for the left-most items in the “simple standing” and “shunning” rows, the top vertices were inadvertently displayed as filled blue circles where they should have been displayed as empty circles.

The correct version of Fig. 2 is:
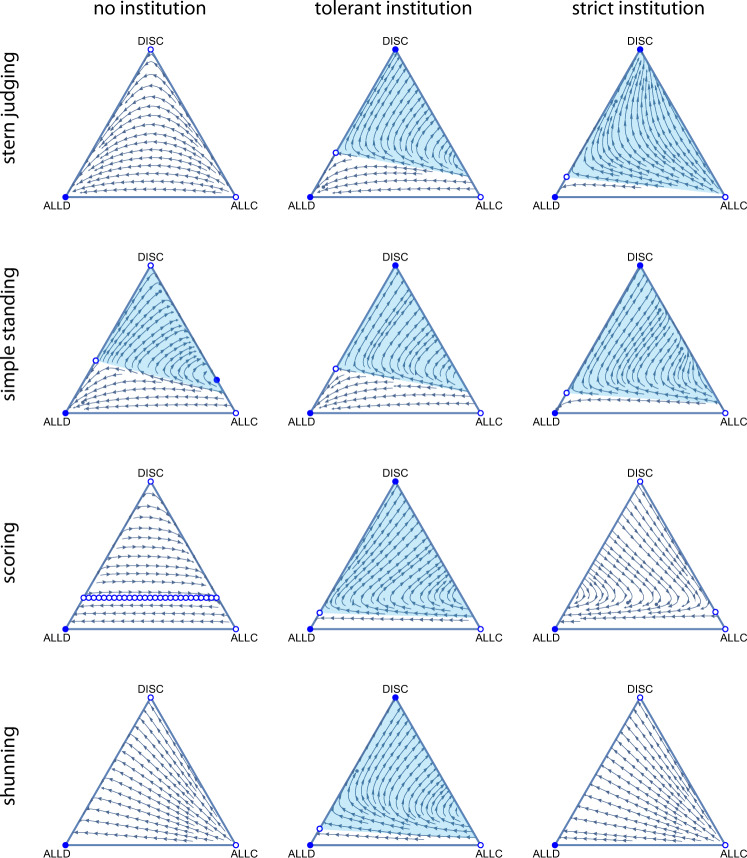


which replaces the previous incorrect version:
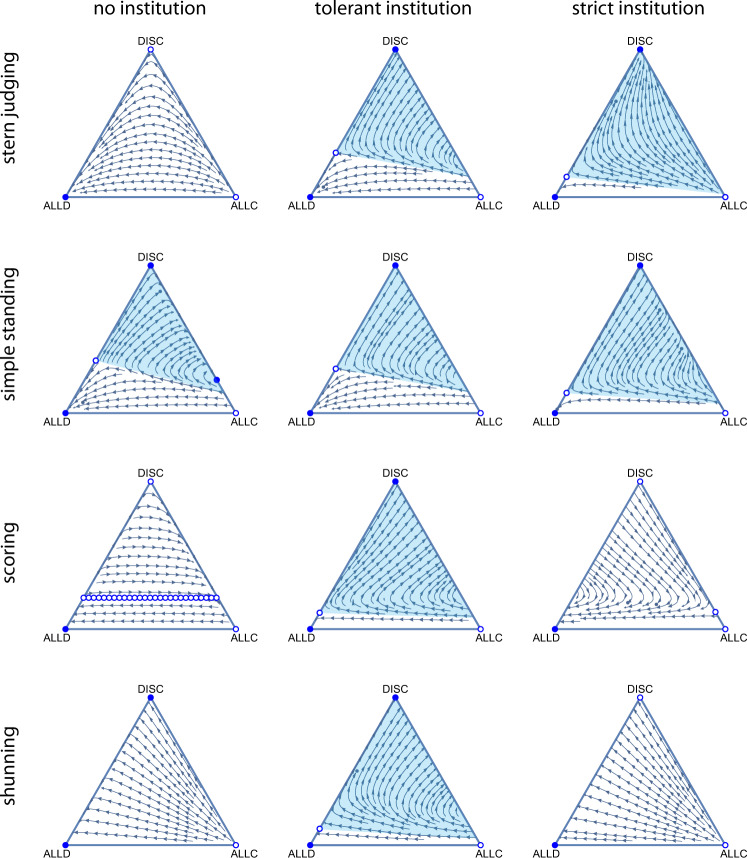


This has been corrected in both the PDF and HTML versions of the Article.

