# Supplementary material for: Adherence to public institutions that foster cooperation
Source: Nat Commun. 2021 Jun 11;12:3567. doi: 10.1038/s41467-021-23783-9 (PMC8196024; doi:10.1038/s41467-021-23783-9)
Supplement: Supplementary file 1 — Supplementary Information [file 41467_2021_23783_MOESM1_ESM.pdf]

Adherence to public institutions that foster cooperation
Supplementary Information

Arunas L. Radzvilavicius[1,*], Taylor A. Kessinger[1,*], Joshua B. Plotkin[1,†]

[1]Department of Biology, University of Pennsylvania, Philadelphia, PA, USA

[*] Equal contribution [†]E-mail: jplotkin@sas.upenn.edu

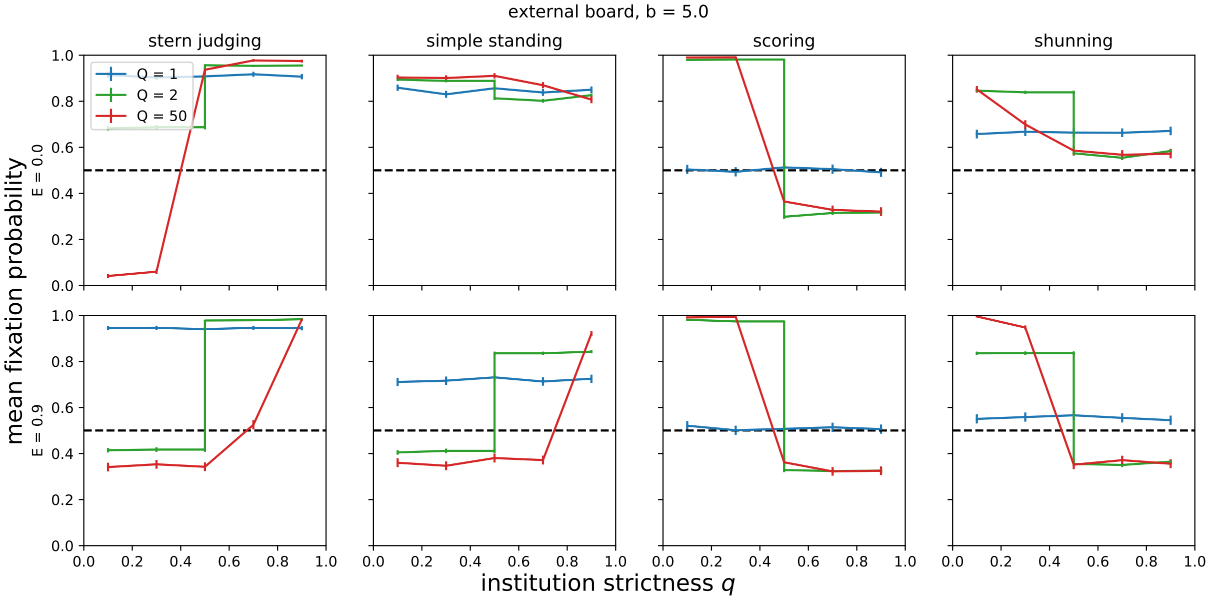

Supplementary Figure 1. Evolution can stabilize adherence to institutions of public monitoring institutions under four social norms. We plot the mean fixation probability of a novel type that follows the institution (DISC-ADHERE) in a population that otherwise uses private assessment (DISC-PRIVATE), averaged over an initial frequency f_{Z_a} drawn uniformly from the open interval $(0, 1)$. Horizontal lines (0.5) indicate the neutral fixation probability. The institution is comprised of an external board. Error bars indicate 95% confidence intervals for the binomial probability of fixation estimated from 2,500 replicate Monte Carlo simulations with population size $N = 50$ and error rates $e_1 = e_2 = 0.02$.

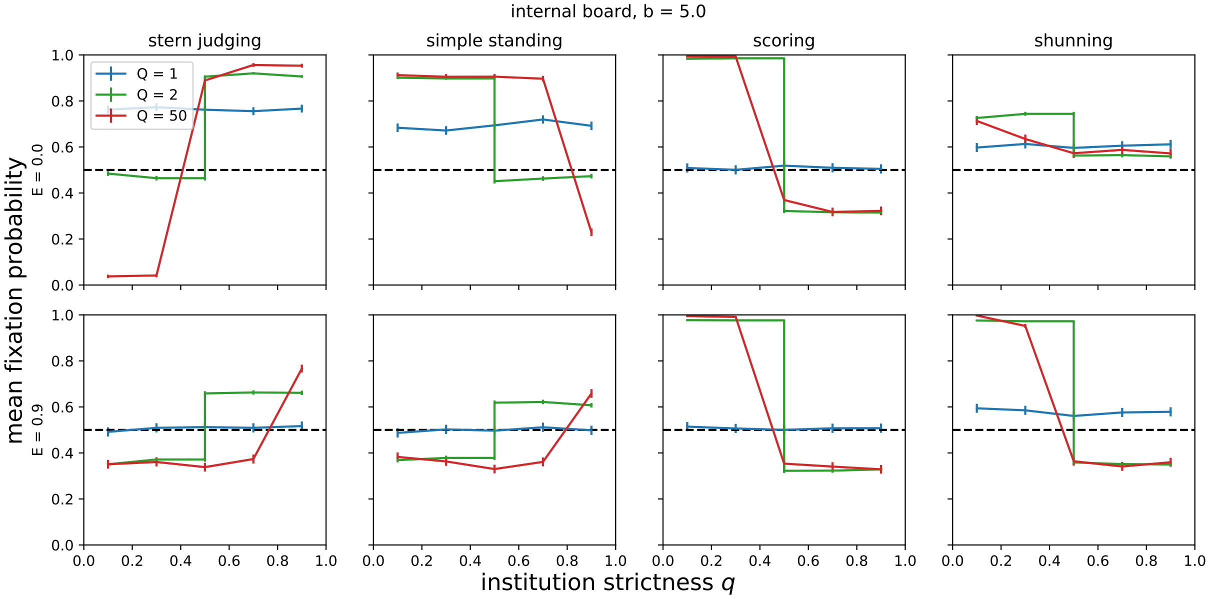

Supplementary Figure 2. Evolution can stabilize adherence to institutions of public monitoring under four social norms. All parameter values and notations and error bars are identical to Supplementary Figure 1, except that in this figure the institution is comprised of an internal board.

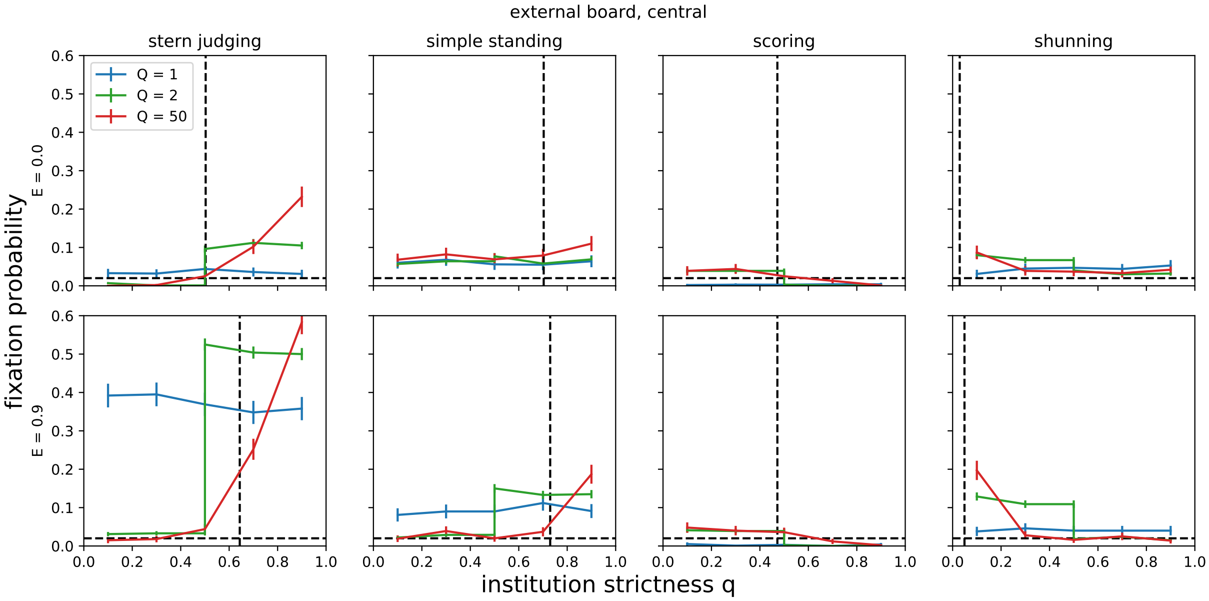

Supplementary Figure 3. Fixation probability for a single institutional adherent (DISC-ADHERE, Z_a) introduced in a population three non-adherent types (ALLC, X; ALLD, Y; and DISC-PRIVATE, Z_n) initially each at frequencies 16/50, 17/50, and 17/50, respectively. Parameter values and error bars are otherwise the same as in Figure 4.

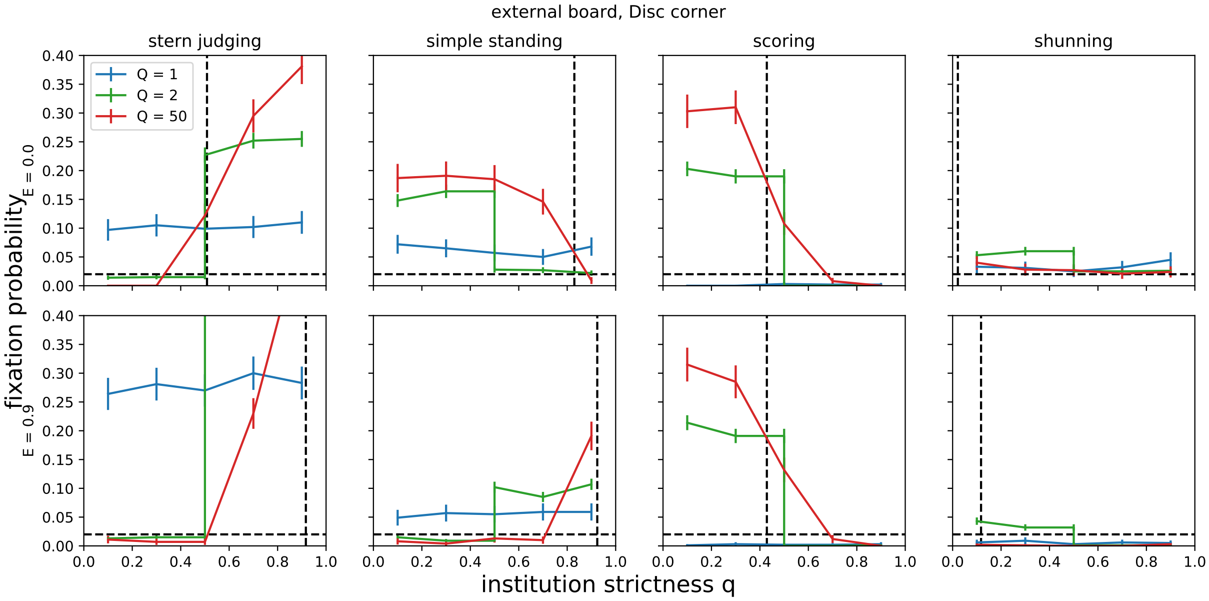

Supplementary Figure 4. Fixation probability for a single institutional adherent (DISC-ADHERE, Z_a) introduced in a population of three non-adherent types (ALLC, X; ALLD, Y; and DISC-PRIVATE, Z_n) with initial frequencies $f_X = 2/50$, $f_Y = 2/50$, and $f_{Z_n} = 46/50$. Parameter values and error bars are otherwise the same as in Figure 4.

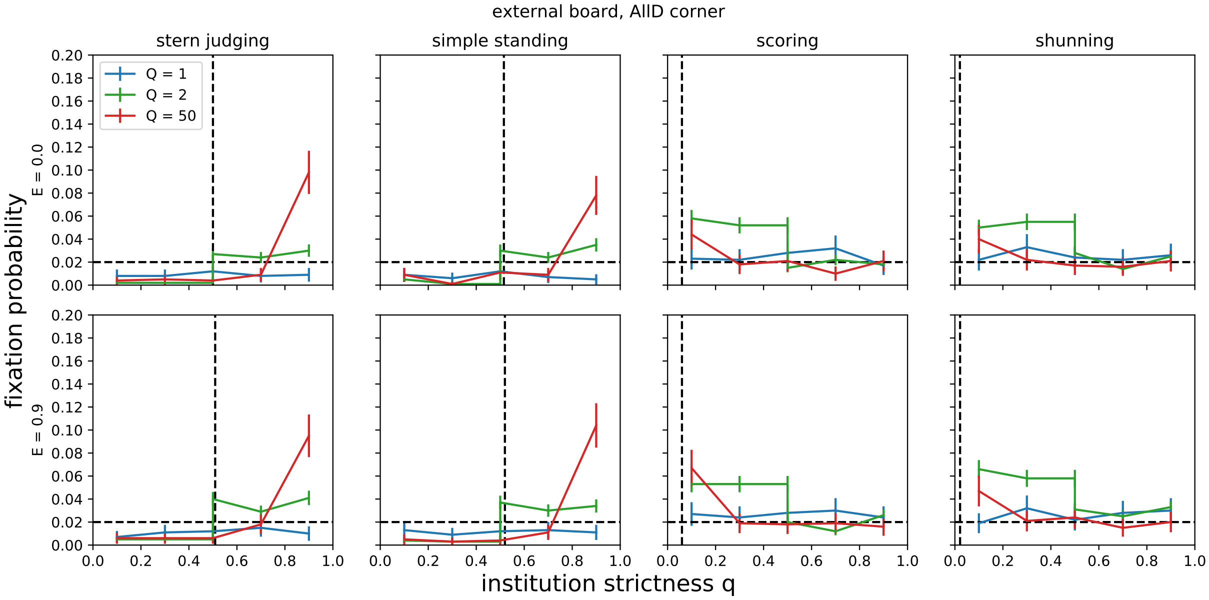

Supplementary Figure 5. Fixation probability for a single institutional adherent (DISC-ADHERE, Z_a) introduced in a population of three non-adherent types ((ALLC, X; ALLD, Y; and DISC-PRIVATE, Z_n) with initial frequencies $f_X = 2/50$, $f_Y = 46/50$, and $f_{Z_n} = 2/50$. Parameter values are otherwise the same as in Figure 4.

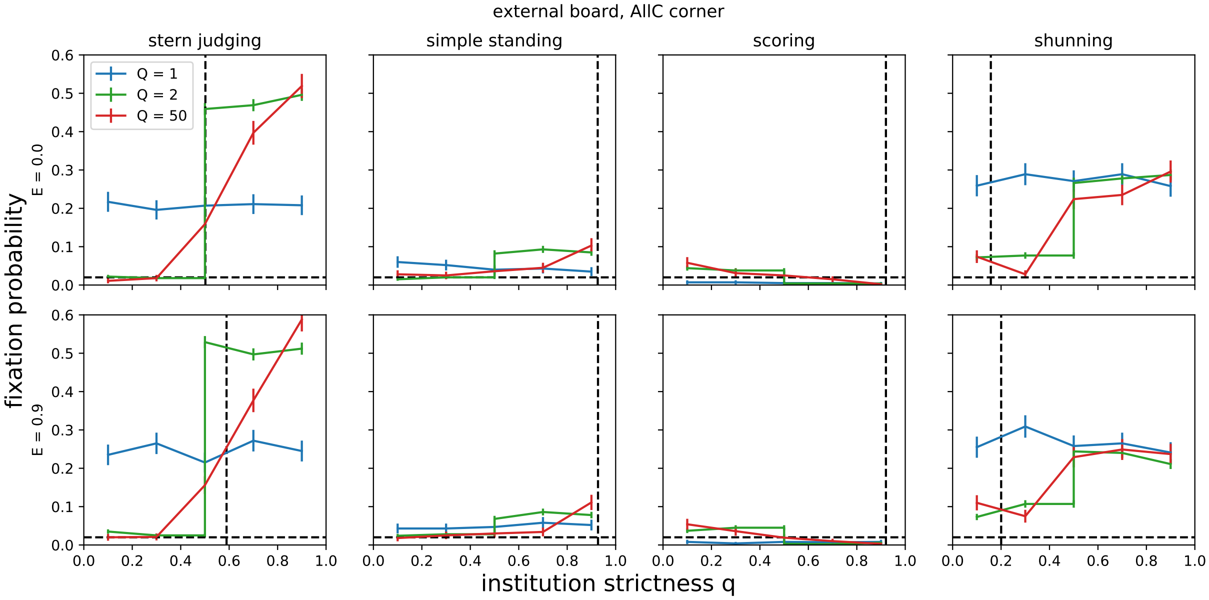

Supplementary Figure 6. Fixation probability for a single institutional adherent (DISC-ADHERE, Z_a) introduced in a population of three non-adherent types (ALLC, X; ALLD, Y; and DISC-PRIVATE, Z_n) with initial frequencies $f_X = 46/50$, $f_Y = 2/50$, and $f_{Z_n} = 2/50$. Parameter values and error bars are otherwise the same as in Figure 4.

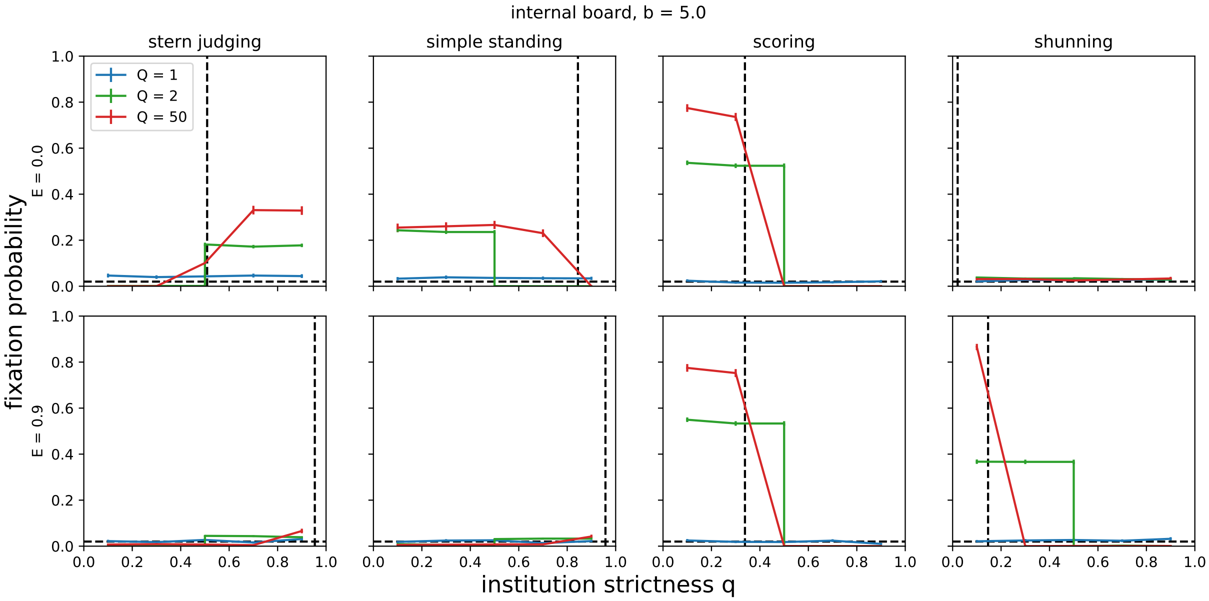

Supplementary Figure 7. Fixation probability for a single institutional adherent (DISC-ADHERE, Z_a) introduced in a population of non-adherents (DISC-PRIVATE, Z_n), with $b = 5$ and an internal board. Parameter values are otherwise the same as in Figure 4.

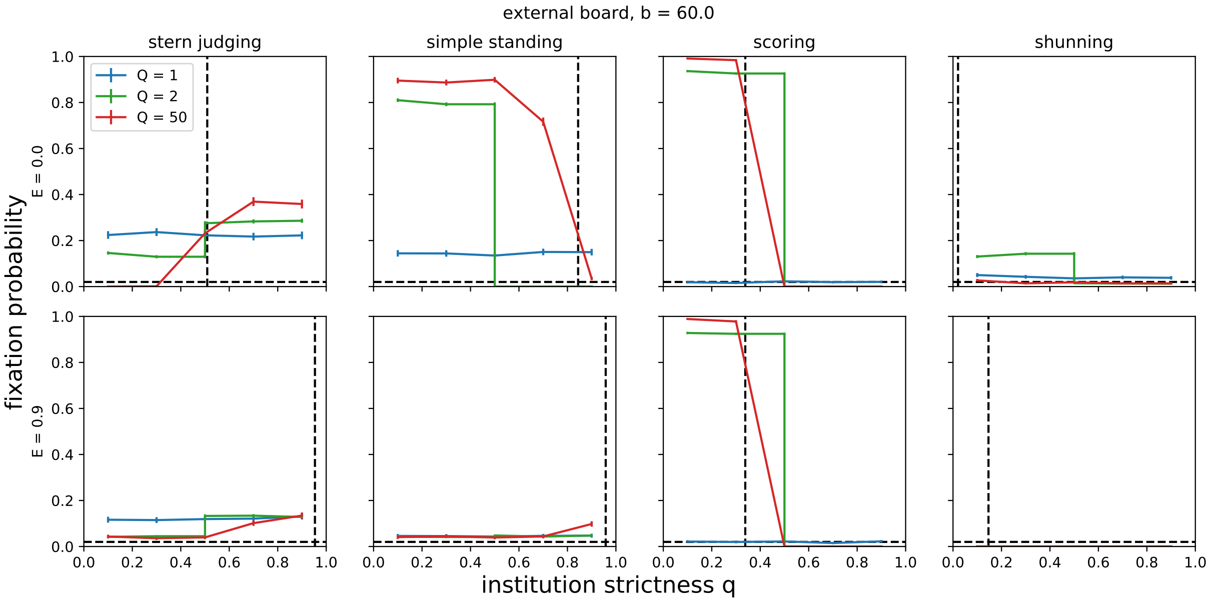

Supplementary Figure 8. Fixation probability for a single institutional adherent (DISC-ADHERE, Z_a) introduced in a population of non-adherents (DISC-PRIVATE, Z_n), with $b = 60$ and an external board. Parameter values and error bars are otherwise the same as in Figure 4.

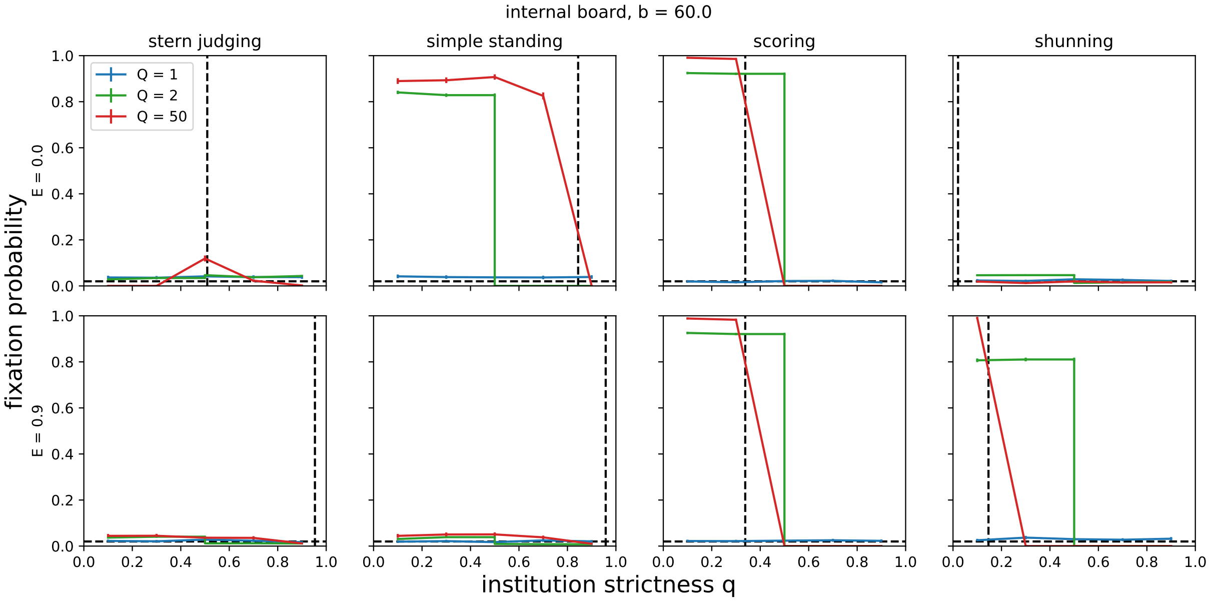

Supplementary Figure 9. Fixation probability for a single institutional adherent (DISC-ADHERE, Z_a) introduced in a population of non-adherents (DISC-PRIVATE, Z_n), with $b = 60$ and an internal board. Parameter values and error bars are otherwise the same as in Figure 4.

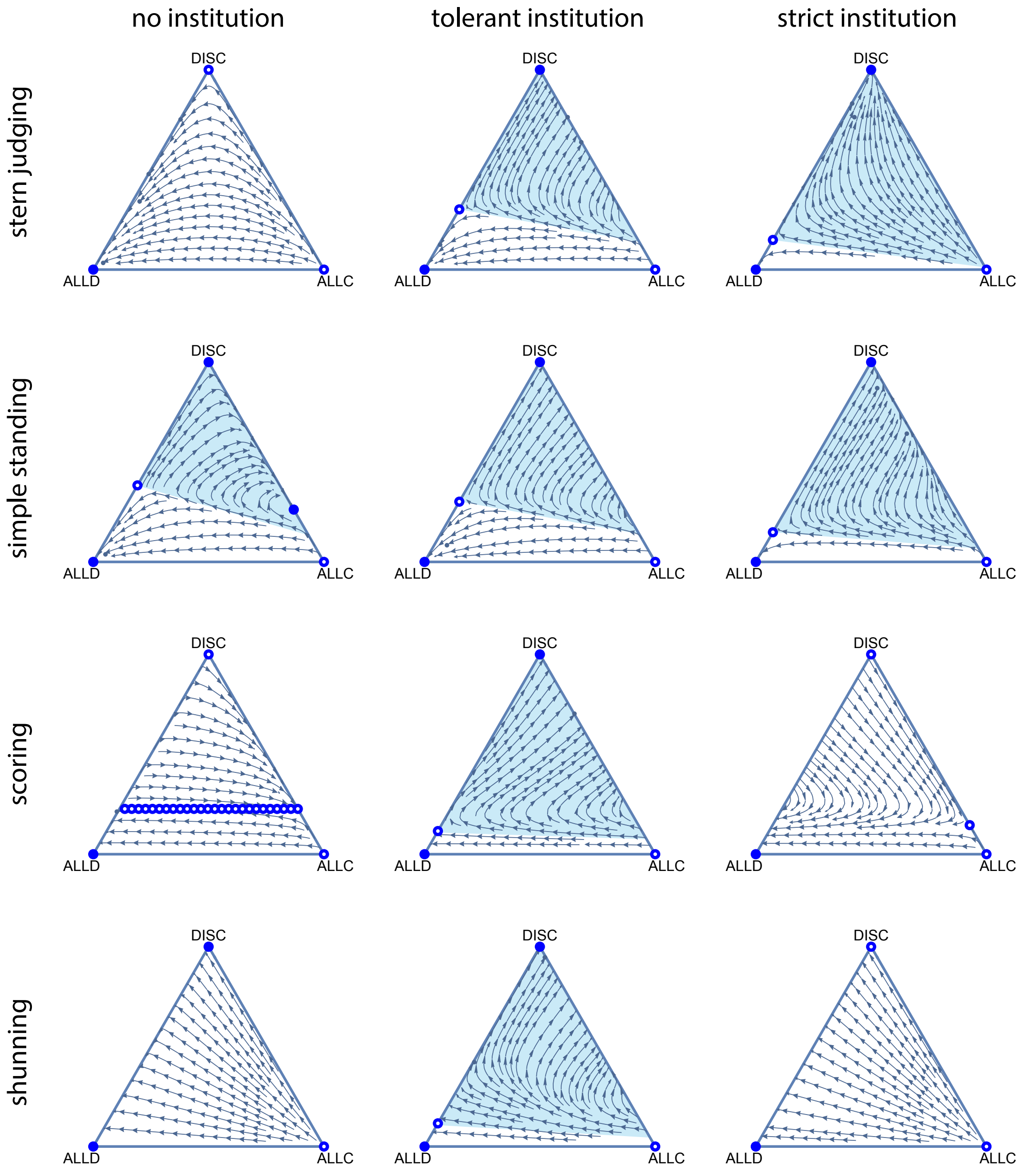

Supplementary Figure 10. Selection gradient of strategy evolution under replicator dynamics. All notation and parameter values are identical to Figure 2, except that in this case e_1 exceeds e_2: $e_1 = 0.1$ and $e_2 = 0.01$.

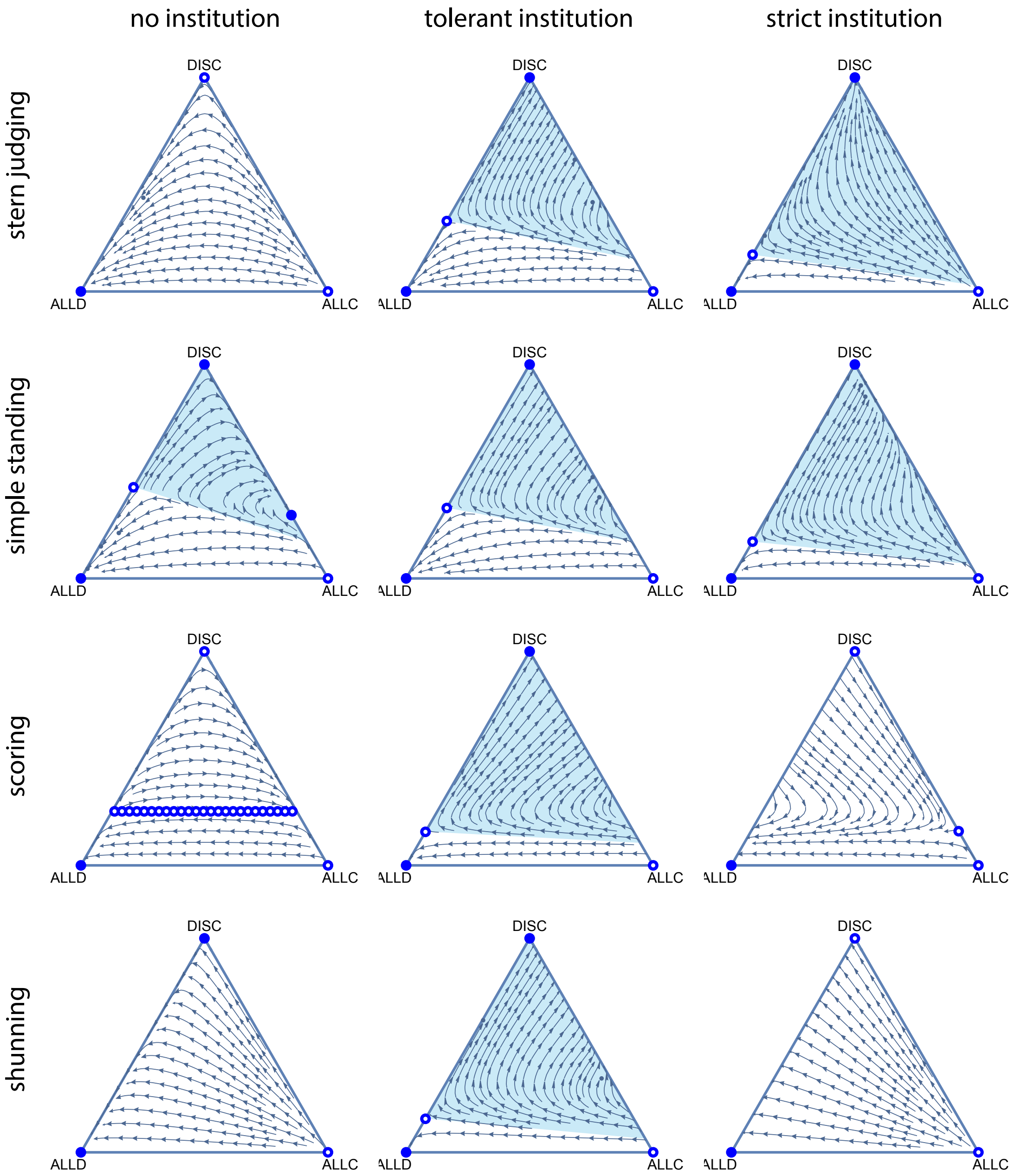

Supplementary Figure 11. Selection gradient of strategy evolution under replicator dynamics. All notation and parameter values are identical to Figure 2, except that in this case e_2 exceeds e_1: $e_1 = 0.01$ and $e_2 = 0.1$.

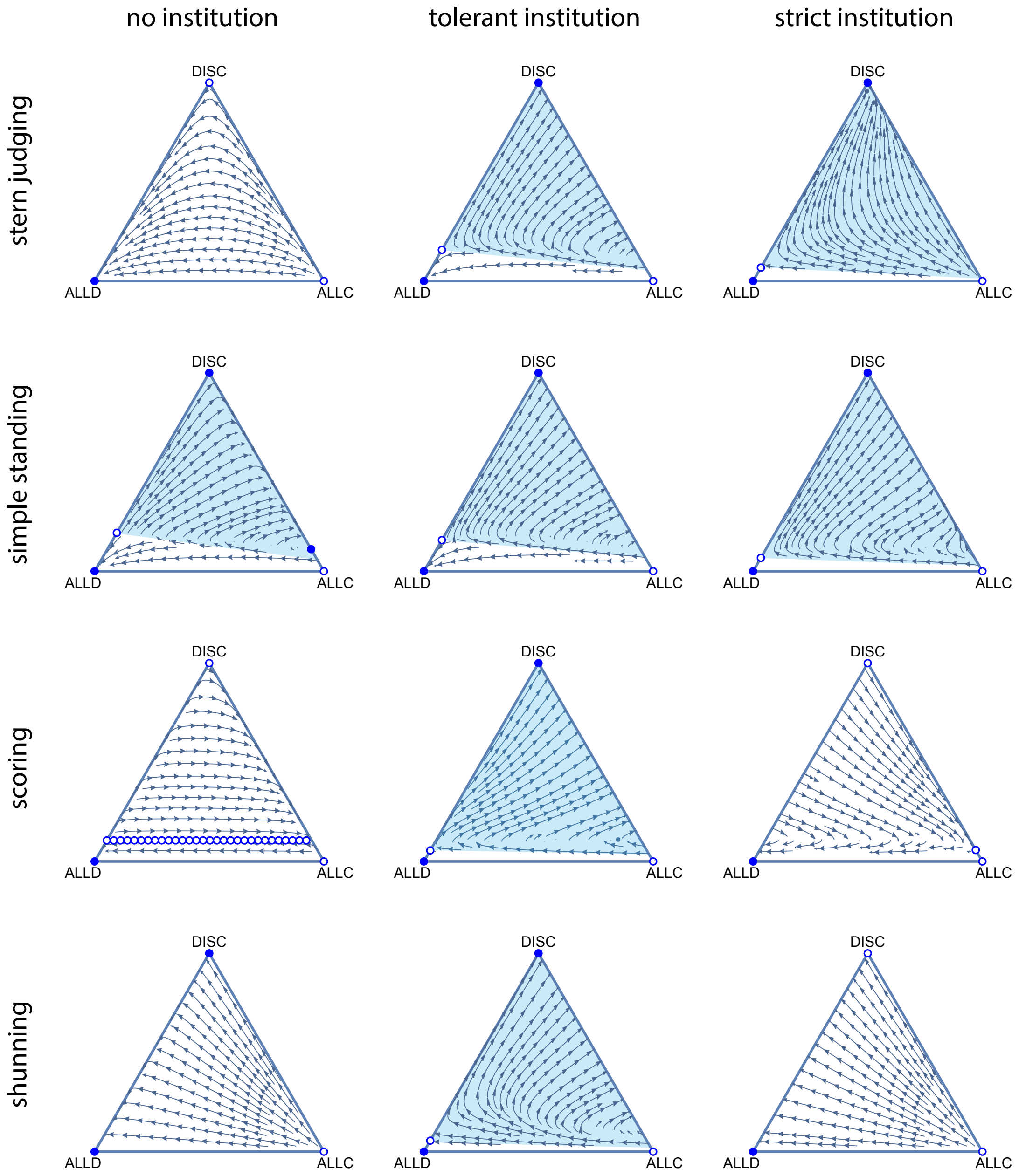

Supplementary Figure 12. Selection gradient of strategy evolution under replicator dynamics. All notation and parameter values are identical to Figure 2, except that in this the benefit of cooperation is larger: $b = 10$.

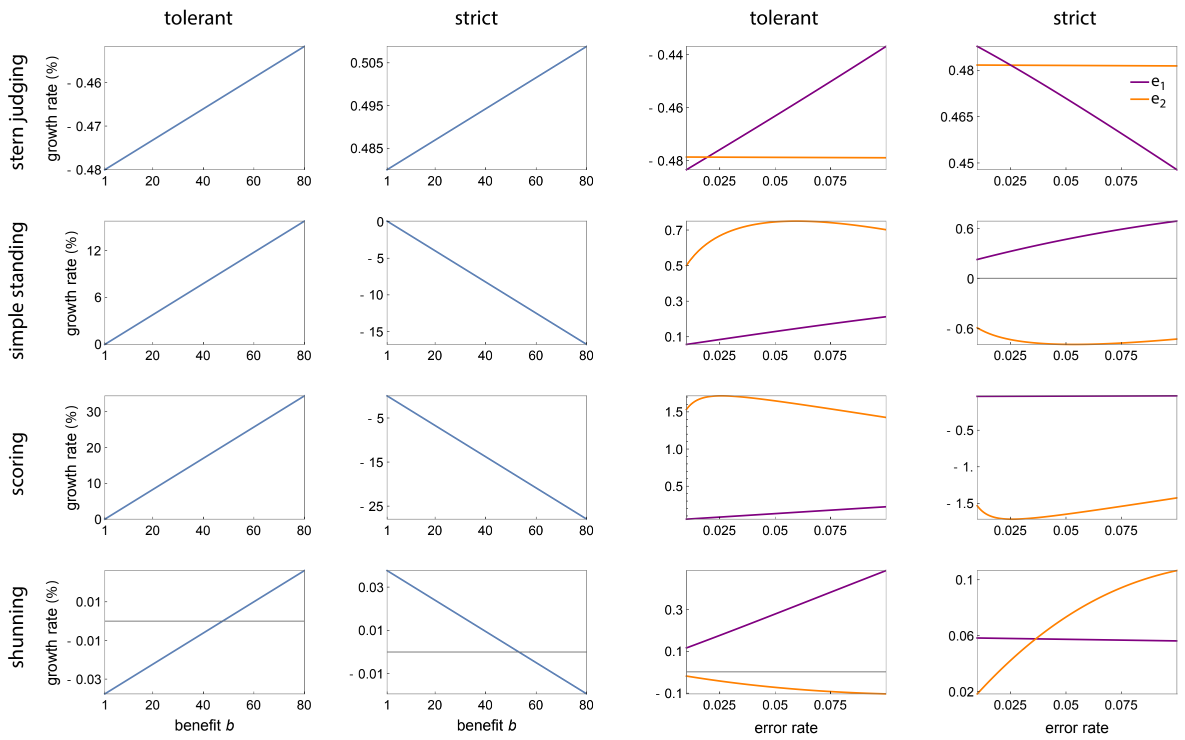

Supplementary Figure 13. Growth rate of institutional adherents (DISC-ADHERE, Z_a) in a population otherwise composed of private assessors (DISC-PRIVATE, Z_n). All parameters and notation are the same as in Figure 5, except that the institution here consists of an internal board.

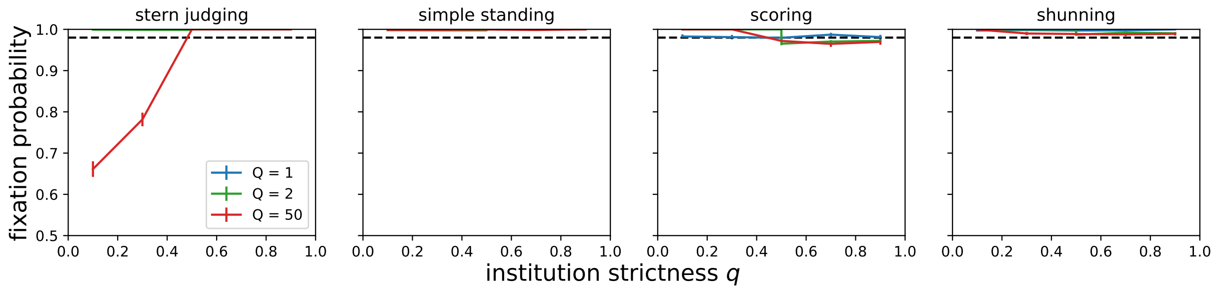

Supplementary Figure 14. The stability of institutional adherents. We initialize a population of institutional adherents (DISC-ADHERE) and allow reputations to equilibriate. We then introduce a single non-adherent (DISC-PRIVATE) and record the number of simulations in which DISC-ADHERE fixes. Fixation probabilities exceeding $1 - 1/N$ (dotted horizontal line) indicate that selection promotes the robustness of institutional adherence against invasion by non-adherents. Error bars indicate 95% confidence intervals for the binomial fixation probability across 2,500 replicate Monte Carlo simulations with population size $N = 50$ and error rates $e_1 = e_2 = 0.02$. The institution is comprised of an external board of Q members.
